# CBX2-dependent transcriptional landscape: implications for human sex development and its defects

**DOI:** 10.1038/s41598-019-53006-7

**Published:** 2019-11-12

**Authors:** Patrick Sproll, Wassim Eid, Anna Biason-Lauber

**Affiliations:** 1 Division of Endocrinology, Section of Medicine, University of Fribourg, Fribourg, 1700 Switzerland; 2 Department of Biochemistry, Medical Research Institute, University of Alexandria, Alexandria, 21526 Egypt

**Keywords:** Transcriptomics, Endocrinology

## Abstract

Sex development, a complex and indispensable process in all vertebrates, has still not been completely elucidated, although new genes involved in sex development are constantly being discovered and characterized. Chromobox Homolog 2 (CBX2) is one of these new additions and has been identified through a 46,XY girl with double heterozygous variants on CBX2.1, causing Differences of Sex Development (DSD). The mutated CBX2.1 failed to adequately regulate downstream targets important for sex development in humans, specifically steroidogenic factor 1 (NR5A1/SF1). To better place CBX2.1 in the human sex developmental cascade, we performed siRNA and CBX2.1 overexpression experiments and created a complete CRISPR/Cas9-CBX2 knockout in Sertoli-like cells. Furthermore, we deployed Next Generation Sequencing techniques, RNA-Sequencing and DamID-Sequencing, to identify new potential CBX2.1 downstream genes. The combination of these two next generation techniques enabled us to identify genes that are both bound and regulated by CBX2.1. This allowed us not only to expand our current knowledge about the influence of CBX2.1 in human sex development, but also to advance our insight in the mechanisms governing one of the most important decisions during embryonal development, the commitment to either female or male gonads.

## Introduction

The moment at which the bipotential gonad commits to either the testicular or the ovarian pathway, is one of the most defining stages during development. It is a subtle difference at first, but it turns into a life-defining state governing large parts of animal behavior and is finally responsible for the continuation of entire species. However important this event is, we still have not decoded the complete blueprint of gene and protein interactions behind it in most animals, including humans. Numerous different players are involved and variants or abnormal expression of any one of them can lead to Differences of Sex Development (DSD) in patients. The analysis of one such DSD case led to the identification of CBX2 as one of these sex development genes. CBX2, also known as Chromobox Homolog 2, is a conserved regulatory factor and a polycomb group (PcG) protein. PcG proteins were discovered in *Drosophila*, where they are involved in maintaining the silent state of Hox genes during development^1^. It could be shown that ablation of M33, the mouse homologue of CBX2, causes male-to-female sex reversal in mice. M33 knockout mice are sterile, and 50% of the Sry positive mice are phenotypically female, with ovarian-like tissue^2^. The work of Katoh-Fukui *et al*. placed M33 upstream of Sry in the murine sex development cascade, due to the timing of expression and affected development of the genital ridges in both XX and XY embryos. Later, they showed that during adrenal and spleen development in mice, M33 is able to activate Sf1 expression, which suggests that M33 not only functions as a chromatin modifier, but is also a transactivator^3^. Interestingly, forced expression of Sry or Sox9 in M33 KO mice is able to rescue the sex reversal in XY mice^4^. However, these mice presented with smaller gonads compared to WT mice, which led Katoh-Fukui *et al*. to conclude that M33 potentially regulates testis determination by directly or indirectly regulating Sry expression, and might play a role in gonadal growth by regulating other factors.

As previously mentioned, a double heterozygote variant on CBX2.1 was discovered in a 46,XY girl who presented with normal female genitalia, uterus and ovarian-like tissue. Transactivation experiments revealed that the variant CBX2.1 does not adequately stimulate the expression of the target gene NR5A1 (also known as SF1), which is essential for human sex development^5^. This placed CBX2.1 upstream of SRY in the human sex development cascade, which is in accordance with the mouse experiments and the known expression window of CBX2 in the early male gonad (week 7 of gestation), prior to testis determination^6^. The expression pattern for CBX2 has been validated using singe cell RNA-Sequencing (scRNA-Seq) from fetal gonads. CBX2 was shown to be expressed from week 9 to week 25 post-fertilization in both male and female gonads^7^. To deepen our understanding of the role of CBX2.1 in human sex development, we also performed genome-wide protein-DNA interaction studies (DamID) coupled with Next Generation Sequencing (NGS). We were able to identify multiple genes bound and regulated by CBX2.1 and hypothesized that CBX2.1 is a stimulator of the male (through SOX9 and NR5A1 stimulation) and an inhibitor for the female pathway (through inhibition of FOXL2 expression)^8^.

CBX2 has a second isoform, CBX2.2, which has very recently been identified as a DSD gene in two unrelated 46,XY patients presenting with complete gonadal dysgenesis^9^. Transactivation studies showed that the variant CBX2.2 failed to regulate the expression of several genes including EMX2, an important factor for bipotential gonadal development.

In order to expand our knowledge on the CBX2.1-dependent transcriptional landscape in human gonad development, we took advantage of NGS and bioinformatics approaches.

## Results

### Identification of CBX2.1 dependent genes

RNA-Seq of human Sertoli-like cells under CBX2.1 knockdown using siRNA and overexpression of wild type CBX2.1 has been performed. The NGS approach returned 2176 significantly regulated genes under CBX2.1 knockdown (Fold Change (FC) > 1, False Discovery Rate (FDR) < 0.05) and 146 significantly regulated genes under CBX2.1 overexpression, 54 of which show a significant change in expression under both treatments. Reproducibility between the same treatments was confirmed by creating a heatmap (The R Project, gplots package) (Supplemental Fig. 1)^10,11^.

### GO-Enrichment analysis

**Figure 1:**
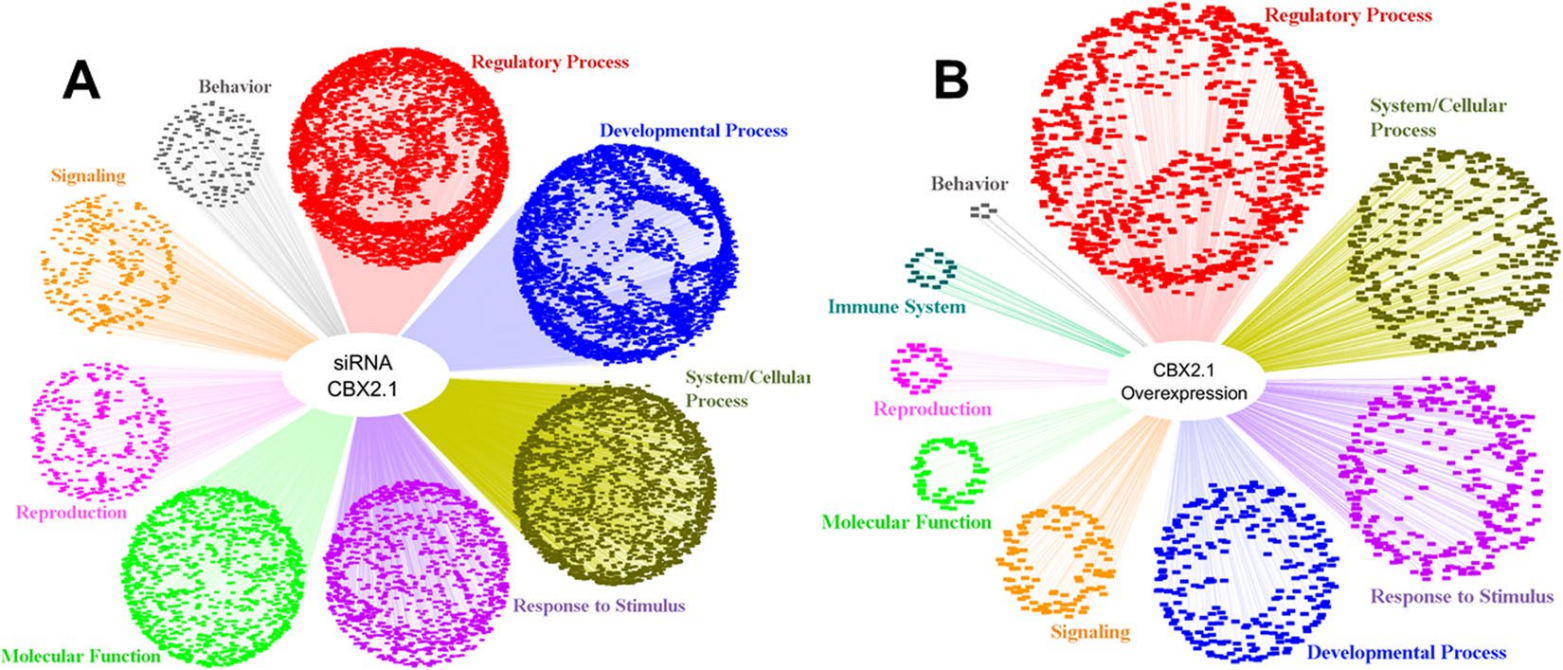
Unbiased GO-enrichment analysis. GO-terms related to Biological Processes have been split into different subcategories (e.g. Regulatory Process, Developmental Process, etc.) while terms related to Molecular Function have been gathered together under this term. (**A**) GO-enrichment for genes that significantly change their expression under CBX2.1 knockdown. (**B**) GO-enrichment for genes that significantly change their expression under CBX2.1 overexpression. Visualization of the data has been performed using Cytoscape 3.7.1.

To gain insights into the general function of the CBX2.1 transcriptional landscape, unbiased Gene Ontology (GO)-enrichment analysis of NGS data was performed (Fig. 1). Under CBX2.1 downregulation, the significantly up- and down-regulated genes are enriched for GO-terms mostly involved in developmental and regulatory processes (Fig. 1A). However, CBX2.1 dependent genes are also involved in various other biological processes like system/cellular processes, response to stimulus, behavior, signaling and reproduction. Of special note is the enrichment of CBX2.1 downstream genes in GO-terms such as Sex Differentiation (GO:0007548), Reproductive System Development (GO:0061458), Development of Primary Sexual Characteristics (GO:0045137), Urogenital System Development (GO:0001655) and Gonad Development (GO:0008406). Additionally, CBX2.1 dependent genes also show enrichment for other developmental process like kidney development, CNS development, spleen development and bone development. This is in accord with what has previously been shown in mice with M33 (CBX2 homologue) ablation, which showed skeletal malformations and defects in the splenic and adrenal development, although human mutants do not seem to recapitulate these defects^3^. In the CBX2.1 overexpression experiments, genes that show a significant regulation are mostly involved in regulatory processes (Fig. 1B), but also response to stimulus, signaling, system/cellular process, developmental process, immune system process, behavior and reproduction. Again, genes which are affected by CBX2.1 overexpression are enriched for the GO-terms Reproductive Structure Development (GO:0048608) and Urogenital System Development (GO:0001655).

### RNA-seq gene expression validation

For the gene expression validation of the CBX2.1 RNA-Seq, we first focused on the genes that showed the most significant change in expression under either CBX2.1 knockdown or overexpression CBX2.1 (FDR < 0.001, Table 1) in an unbiased approach. When endogenous CBX2.1 was downregulated, HORMA Domain Containing 1 (HORMAD1), ELAV Like Neuron-Specific RNA Binding Protein 3 (ELAVL3), NK2 Homeobox 4 (NKX2-4), were upregulated (FC of 10.8, 6.1 and 4.3, respectively), while Adenylate Kinase 4 (AK4), Tight Junction Protein 2 (TJP2) and Ubiquitination Factor E4B (UBE4B) were downregulated (FC 0.14, 0.12 and 0.1, respectively). Real-time PCR (qRT-PCR) was used to validate the expression of these genes. Under overexpression of CBX2.1, the highest regulated targets identified by RNA-Seq were Growth Hormone 1 (GH1) and Claudin 4 (CLDN4), with a fold-change (FC) of 17.9 and 8.1 respectively.

**Table 1:** Genes presenting with the highest change of expression under either CBX2.1 knockdown or CBX2.1 overexpression.

Treatment	Gene	RNA-Seq (FC)	qRT-PCR (rel. Exp.)
CBX2.1 siRNA	HORMAD1	10.8	8.1*
	ELAVL3	6.1	1.8*
	NKX2-4	4.3	—
	AK4	0.14	—
	TJP2	0.12	0.08****
	UBE4B	0.1	0.09***
CBX2.1 overexpression	GH1	17.9	1.5*
	CLDN4	8.1	—

The values for the RNA-Seq are represented as fold-change (FC), indicating the expression change of the target gene compared to its expression under endogenous CBX2.1 expression (i.e. EV transfection or scrambled siRNA transfection). The values for the RT-qPCR show the results from the validation experiments as relative expression values (2^−ΔΔCt^) compared to the control. The error is presented as the standard error of the mean (SEM) and unpaired t-test was performed to calculate the significance. (****) P < 0.0001; (***) P < 0.001; (*) P < 0.05.

**Figure 2:**
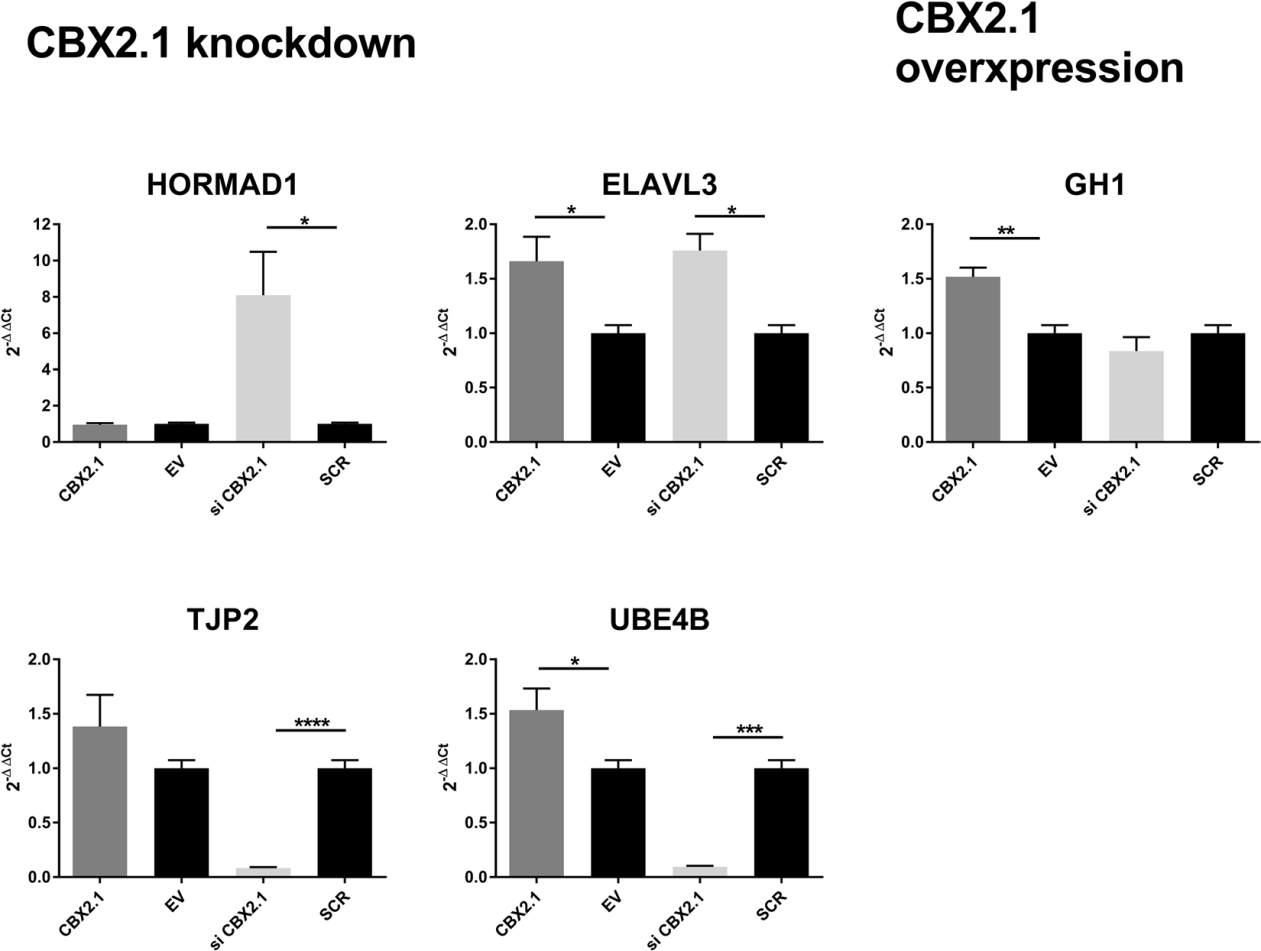
RT-qPCR quantification of mRNA levels for putative CBX2.1 expression dependent targets. NT2-D1 cells transfected with siRNA against endogenous CBX2.1 (si CBX2.1), scrambled siRNA (SCR), WT CBX2.1 or an empty vector (EV). The relative expression levels (2^−ΔΔCt^) of the target genes were calculated by normalization with cyclophilin expression as an endogenous control. The data in all graphs are the average of at least three independent experiments, error bars represent standard error of the mean (SEM) and values are expressed as relative to the control, e.g. either EV = 1 or SCR = 1. (****) P < 0.0001; (***) P < 0.001; (**) P < 0.01; (*) P < 0.05.

The effect of CBX2.1 could be validated for five of the eight selected CBX2.1 dependent genes, albeit not at the same levels seen in the RNA-Seq experiments (Fig. 2). The CBX2.1 knockdown targets HORMAD1 and ELAVL3 were upregulated (8.1 and 1.8, respectively; P < 0.05) and TJP2 and UBE4B downregulated (0.08 and 0.09, respectively; P < 0.001). GH1 was upregulated 1.5-fold (P < 0.01) by CBX2.1 overexpression, compared to control. Furthermore, ELAVL3 and UBE4B are also significantly upregulated when CBX2.1 was overexpressed, which has not been observed by the RNA-Seq. Due to very low basal expression, the change of expression for the three genes, CLDN4, NKX2-4 and AK4 could not be validated.

**Figure 3:**
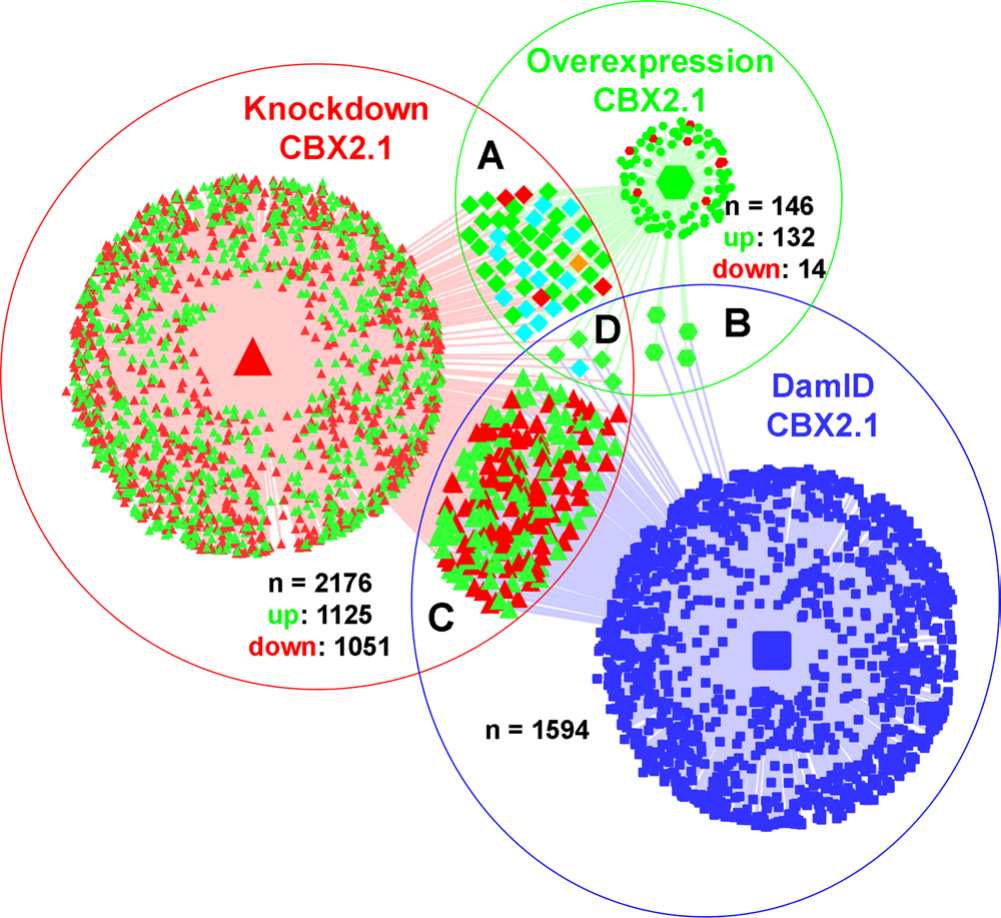
Venn diagram comparing gene regulation. Comparing genes that are significantly regulated under CBX2.1 overexpression (green-circle), CBX2.1 knockdown (red-circle) and genes bound by CBX2.1 according to DamID (blue-circle). (**A**) 54 genes regulated under both CBX2.1 overexpression and downregulation. (**B**) 4 genes regulated under CBX2.1 overexpression and bound by CBX2.1. (**C**) 224 genes regulated under CBX2.1 knockdown and bound by CBX2.1. (**D**) Genes bound by CBX2.1 and regulated under both CBX2.1 overexpression and knockdown. Upregulated genes are colored in green, downregulated genes in red, genes upregulated under CBX2.1 knockdown and downregulated under CBX2.1 overexpression in orange, genes downregulated under CBX2.1 knockdown and upregulated under CBX2.1 overexpression in cyan and genes bound by CBX2.1 but not influenced by its expression in blue. Cytoscape 3.7.1 was used for visualization.

After the unbiased validation, both direct and indirect CBX2.1 dependent genes were selected based on their known involvement in sexual development. We set to identify direct CBX2.1 dependent genes (previously identified by DamID-Sequencing, n = 1594) that are also up or downregulated by CBX2.1 (RNA-Seq of knockdown and overexpression experiments, n = 2176 and n = 146, respectively) (Fig. 3)^7^. Of the 224 genes present in both DamID and RNASeq knockdown, 113 were up- and 111 downregulated. Nine genes were found in the DamID and RNASeq overexpression data, all of which were upregulated. Finally, 54 genes are present in both transcriptome experiments, but not in the DamID data set.

**Figure 4:**
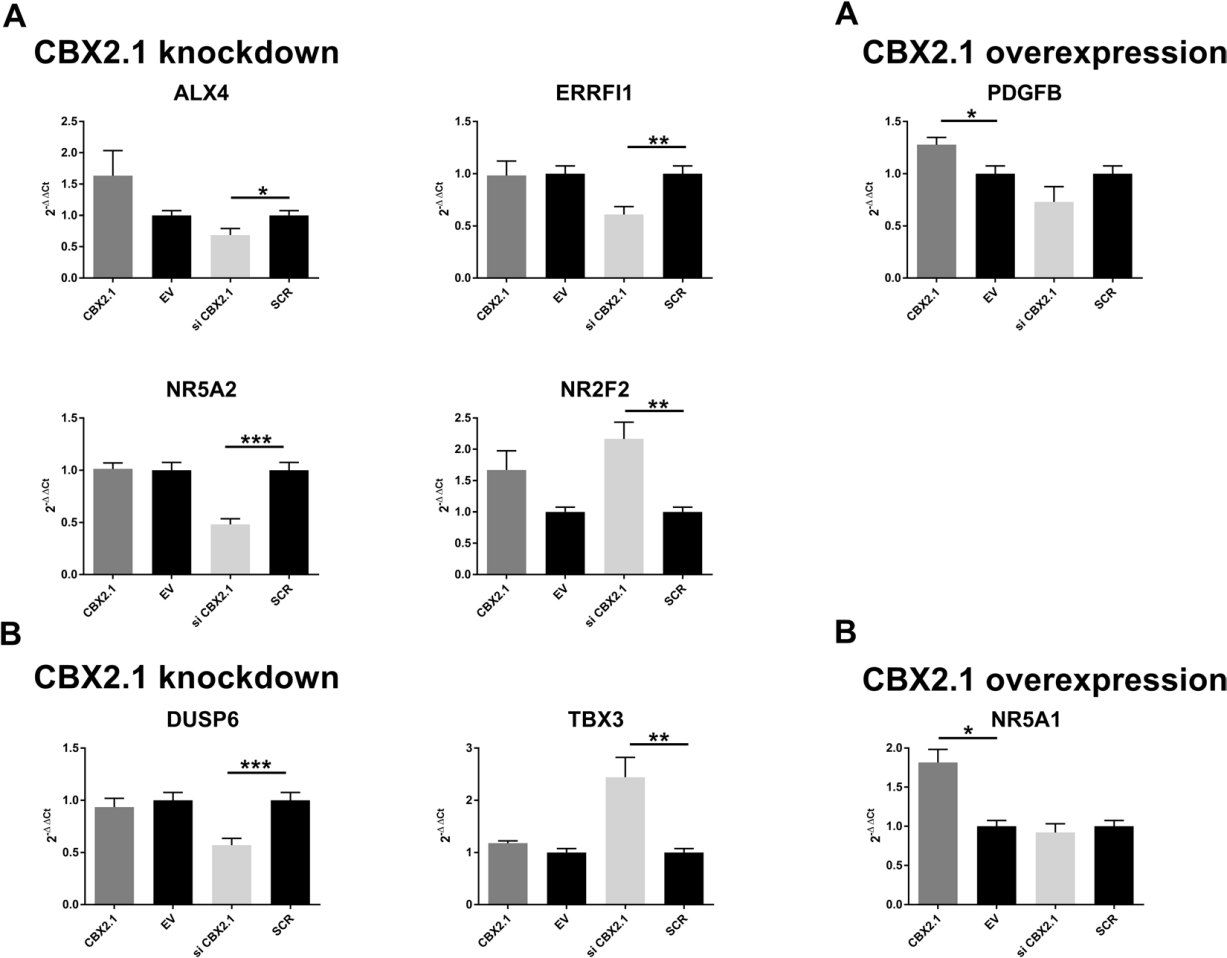
RT-qPCR quantification for gene influenced by CBX2.1 expression and selected based on their involvement in sex development. (**A**) Genes directly bound by CBX2.1. (**B**) Indirect targets of CBX2.1. NT2-D1 cells were transfected with WT CBX2.1, empty vector (EV), siRNA against endogenous CBX2.1 (si CBX2.1) or scrambled siRNA (SCR). The relative expression levels (2^−ΔΔCt^) of the target genes were calculated by normalization with cyclophilin expression as an endogenous control. The data in all graphs are the average of at least three independent experiments, error bars represent standard error of the mean (SEM) and values are expressed as relative to the control, i.e. either EV = 1 or SCR = 1. (***) P < 0.001; (**) P < 0.01; (*) P < 0.05.

Of the genes known to be bound and influenced by CBX2.1 expression, 12 were selected for further analysis using RT-qPCR (Table 2, Fig. 4). Of these, seven genes show an increased expression under knockdown: ALX Homebox 4 (ALX4), LIM Homeobox 8 (LHX8), FEZ Family Zinc Finger 1 (FEZF1), Nuclear Receptor Subfamily 2, Group F, Member 2 (NR2F2), Pre-B-Cell Leukemia Homeobox 1 (PBX1), Protein Kinase, X-Linked (PRKX) and Sex Determining Region Y 5 (SOX5) (FC: 2.4, 2.8, 1.8, 2.2, 1.6, 1.4 and 1.5, respectively). Four genes show a downregulation under CBX2.1 knockdown: ERBB Receptor Feedback Inhibitor 1 (ERRFI1), Nuclear Receptor Subfamily 5, Group A, Member 2 (NR5A2), Platelet Derived Growth Factor Beta Polypeptide (PDGFB) and POU Class 2 Homeobox 1 (POU2F1) (FC: 0.6, 0.6, 0.6 and 0.8, respectively). Finally, one selected gene Transforming Growth Factor Beta 2 (TGFB2), was upregulated under CBX2.1 overexpression (FC: 1.7). RT-qPCR validation confirmed the significant effect of CBX2.1 expression on five of the selected 12 genes. ALX4, ERRFI1 and NR5A2 were significantly downregulated under CBX2.1 knockdown, while NR2F2 was significantly upregulated (0.7, 0.6, 0.5 and 2.2, respectively; P < 0.05). Under CBX2.1 overexpression, PDGFB was significantly upregulated (1.3; P < 0.05) (Fig. 4A).

**Table 2:** Direct targets of CBX2.1 involved in sex development.

Treatment	Gene	RNA-Seq (FC)	qRT-PCR (rel. Exp.)
CBX2.1 siRNA	LHX8	2.8	1.5
	ALX4	2.4	0.7*
	NR2F2	2.2	2.2*
	FEZF1	1.8	1.1
	PBX1	1.6	1.2
	SOX5	1.5	1.1
	PRKX	1.4	1.1
	POU2F1	0.8	0.8
	ERRFI1	0.6	0.6*
	NR5A2	0.6	0.5*
	PDGFB	0.6	0.7
CBX2.1 overexpression	TGFB2	1.7	0.9

The values for the RNA-Seq are represented as fold-change (FC), indicating the expression change of the target gene compared to its expression under endogenous CBX2.1 expression (i.e. EV transfection or scrambled siRNA transfection). The values for the RT-qPCR show the results from the validation experiments as relative expression values (2^−ΔΔCt^) compared to the control. The error is presented as the standard error of the mean (SEM) and unpaired t-test was performed to calculate the significance. (*) P < 0.05.

Additionally, four genes influenced indirectly by CBX2.1 expression were selected: T-Box 3 (TBX3, FC: 2.6) and Dual Specificity Phosphatase 6 (DUSP6, FC: 0.4), which are affected by CBX2.1 knockdown and Nuclear Receptor Subfamily 5, Group A, Member 1 (NR5A1, FC: 3.7) and Frequently Rearranged In Advanced T-Cell Lymphomas 1 (FRAT1, FC: 0.4), which both show an expression change under CBX2.1 overexpression (Table 3). Expression studies followed by RT-qPCR confirmed the significant downregulation of DUSP6 and upregulation of TBX3 under CBX2.1 knockdown, as well as the significant upregulation of NR5A1 by CBX2.1 overexpression (0.6, 2.4 and 1.8, respectively; P < 0.05) (Fig. 4B).

**Table 3:** Indirect targets of CBX2.1 involved in sex development.

Treatment	Gene	RNA-Seq (FC)	qRT-PCR (rel. Exp.)
CBX2.1 siRNA	TBX3	2.6	2.4**
	DUSP6	0.4	0.6***
CBX2.1 overexpression	NR5A1	3.7	1.8*
	FRAT1	0.4	0.9

The values for the RNA-Seq are represented as fold-change (FC), indicating the expression change of the target gene compared to its expression under endogenous CBX2.1 expression (i.e. EV transfection or scrambled siRNA transfection). The values for the RT-qPCR show the results from the validation experiments as relative expression values (2^−ΔΔCt^) compared to the control. The error is presented as the standard error of the mean (SEM) and unpaired t-test was performed to calculate the significance. (***) P < 0.001; (**) P < 0.01; (*) P < 0.05.

### Complete CBX2 knockout (CRISPR/Cas9)

**Figure 5:**
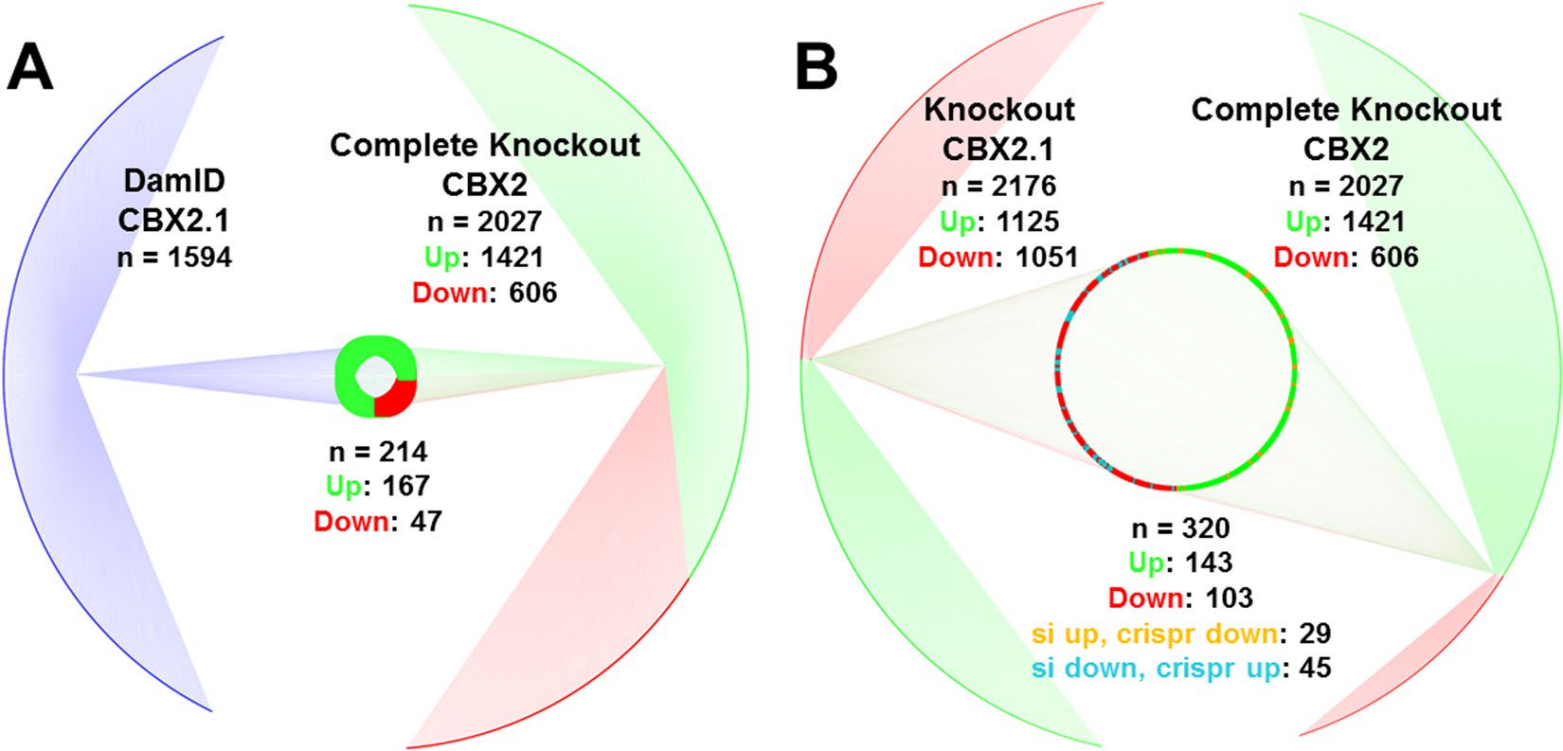
NGS target comparisons. (**A**) Comparison between CRISPR/Cas9 RNA-Seq targets with DamID-Seq targets of CBX2.1. Significantly upregulated targets under CBX2 complete knockdown are colored in green, downregulated targets in red. Genes, which are boundby CBX2.1, are coloured in blue. (**B**) Comparison between CRISPR/Cas9 RNA-Seq data (Complete Knockout) with the CBX2.1 knockdown RNA-Seq data (Knockdown CBX2.1). Significantly upregulated targets are colored in green, downregulated targets in red, genes that are upregulated under CBX2.1 siRNA and downregulated under complete CBX2 knockout are colored in orange and genes that are downregulated under CBX2.1 siRNA and upregulated under CBX2 knockout are colored in cyan. The comparisons were visualized using Cytoscape 3.7.1.

As mentioned, CBX2 has two isoforms, partly distinct in sequence and functions^9,12^. In order to create a Sertoli cell line with a stable CBX2 knockout, durably void of both CBX2 isoforms, a CRISPR/Cas9 construct targeting exon1 was transfected into NT2-D1 cells. The complete knockout was confirmed by western blot and immunofluorescence (Supplemental Fig. 2A,B). RNA-Seq allowed for the identification of 2027 significantly (FDR < 0.01 and FC > 1) regulated genes in CBX2 KO cells, compared to the NT2-D1 cells transfected with CRISPR/Cas9 vector with a scrambled guiding RNA (CRISPR-EV). Of these significantly regulated genes, 1421 were upregulated under complete CBX2 knockout, while 606 were downregulated. By comparing the 2027 significantly regulated genes from the RNA-Seq, with the previously mentioned CBX2.1 DamID-Seq data, 214 genes were identified that are directly regulated by CBX2.1 (Fig. 5A). Of these genes bound by CBX2.1, 167 show an upregulation after complete CBX2 knockout and 47 show a downregulation. We also compared the RNA-Seq data of the siRNA against CBX2.1 and the complete CBX2 knockout RNA-Seq (Fig. 5B). Of the 2027 genes that change expression under complete CBX2 knockout und the 2176 genes that change expression under transient knockdown of CBX2.1 using siRNA, 320 are common between the two. Of which 143 genes are upregulated in both sets, 103 are downregulated in both, 45 are downregulated in the CBX2.1 knockdown and upregulated in the CBX2 knockout and 29 are upregulated in the CBX2.1 knockdown and downregulated in the CBX2 knockout.

The RNA-Seq also allowed for the identification of an insertion of an Adenine (A) on exon1 of CBX2, which leads to a frameshift resulting in a premature stop on exon2 (Supplemental Fig. 3).

### GO-Enrichment analysis

**Figure 6:**
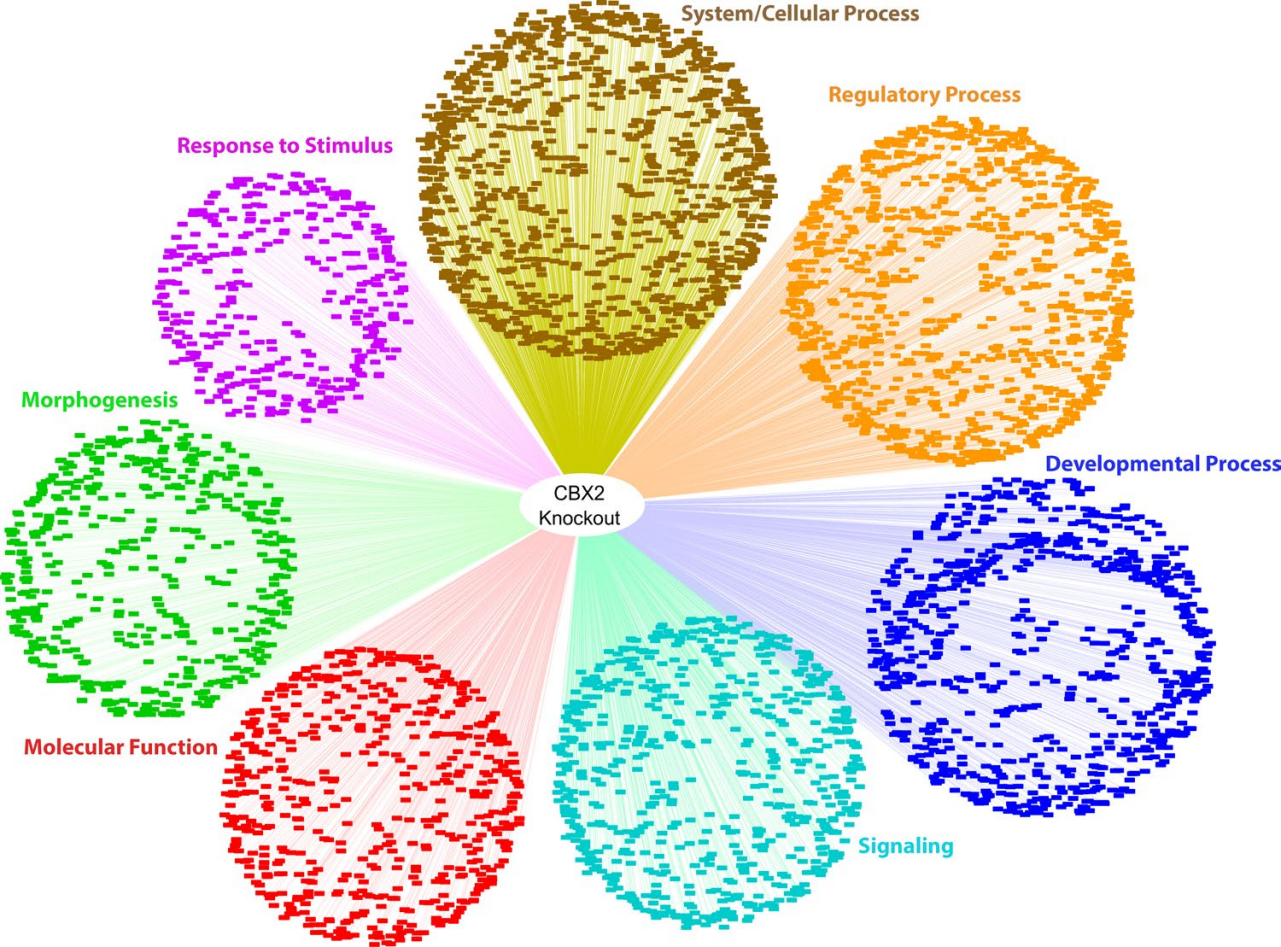
Unbiased GO-enrichment analysis of all significantly regulated genes under complete CBX2 knockout. GO-terms related to Biological Processes have been split into different subcategories (e.g. Regulatory Process, Developmental Process, etc.), while terms related to Molecular Function have been gathered together under this term. Visualization of the data has been performed using Cytoscape 3.7.1.

Genes significantly regulated under CBX2 complete knockout in NT2-D1 cells are involved in various processes, such as system/cellular process, regulatory process and developmental process (Fig. 6). Of particular interest are the GO-enrichments in Urogenital System Development (GO:0001655), Endocrine System Development (GO:0035270), Reproductive System Development (GO:0061458) and Developmental Process Involved in Reproduction (GO:0003006). Again, these enrichments of the unbiased NGS data serve as a proof of concept, illustrating that CBX2, through its downstream targets, is involved in sex development. Similar to the GO-enrichment for the CBX2.1 knockdown, genes of complete CBX2 knockout also show enrichment for kidney development, CNS development and skeletal system development. As previously mentioned, this is in accord with the resulting phenotype in mice with M33 ablation^2,3^.

### RNA-Seq gene expression validation

**Figure 7:**
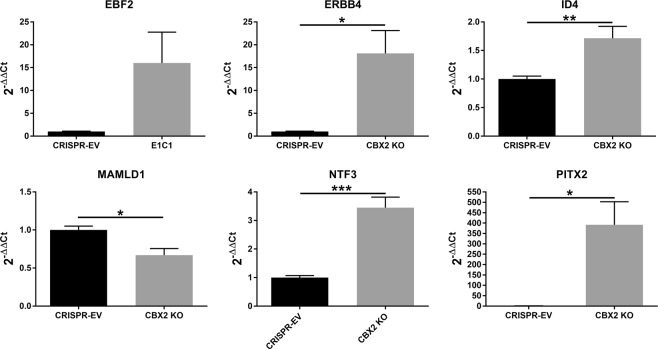
qRT-PCR quantification of selected genes directly bound by CBX2.1. The relative expression values (2^−ΔΔCt^) were calculated compared to the control (CRISPR-EV = 1). The error is presented as the standard error of the mean (SEM) and unpaired t-test was performed to calculate the significance. (***) P < 0.001; (**) P < 0.01; (*) P < 0.05.

Of the 2027 significantly regulated genes under CBX2 complete knockout, 6 were selected for further validation, based on their influence in sex development or possible links to DSD (Table 4). The six selected genes are directly bound by CBX2.1: Early B-Cell Factor 2 (EBF2, FC: 4.8), Erb-B2 Receptor Tyrosine Kinase 4 (ERBB4, FC: 5.3), Inhibitor of DNA Binding 4 (ID4, FC: 2.0), Mastermind Like Domain Containing 1 (MAMLD1, FC: 0.48), Neurotrophin 3 (NTF3, FC: 3.6) and Paired Like Homeodomain 2 (PITX2, FC: 52.9). Expression validation of the genes using qRT-PCR showed that ERRB4, ID4, NTF3 and PITX2 were significantly upregulated in the CBX2 KO cells to a relative expression (2^−ΔΔCt^) of 18.1, 1.7, 3.4 and 391.8, compared to the CRISPR-EV cells, while MAMLD1 was significantly downregulated to a relative expression of 0.7 (Fig. 7). EBF2 was insignificantly (P > 0.05) upregulated to a relative expression level of 16.

**Table 4:** Selected direct targets of CBX2.1.

Gene	RNA-Seq (FC)	qRT-PCR (rel. Exp.)
PITX2	52.9	391.8*
ERBB4	5.3	18.1*
ID4	2.0	1.7*
NTF3	3.6	3.4***
EBF2	4.8	16.0
MAMLD1	0.48	0.7*

The RNA-Seq results for the direct CBX2 targets are shown as fold-change (FC), comparing the CBX2 KO cells with the CRISPR-EV transfected cells. The values for the RT-qPCR show the results from the validation experiments as relative expression values (2^−ΔΔCt^) compared to the controls. The error is presented as the standard error of the mean (SEM) and unpaired t-test was performed to calculate the significance. (***) P < 0.001; (**) P < 0.01; (*) P < 0.05.

## Discussion

In human sexual determination and differentiation, new discoveries are steadily being made. This has important implications, especially for patients with DSD, where the underlying genetic cause is unknown in around half of the cases. The correlation between phenotype and genotype in DSD is particularly challenging, because the phenotype between patients with variants in the same gene can greatly differ. It is therefore important to further study DSD-related genes and expand their interaction networks.

To this end, we choose to gain further insights into the CBX2.1-dependent transcriptional landscape using high throughput genome-wide NGS approaches in a cell model as a surrogate for testicular Sertoli cells. The unbiased data analysis using GO-enrichment showed that CBX2.1 downstream targets are mostly involved in regulatory and developmental process. The analysis also confirmed CBX2′s role in human sex development, since the transcriptionally dependent genes are enriched for GO-terms like Reproductive System Development, Reproductive Structure Development, Gonad Development, and Sex Differentiation (Figs 1 and 6). M33 deficient mice, besides defects in gonadal development, also present with skeletal malformations and defects in splenic and adrenal development^2,3^. However, although CBX2 downstream targets are enriched for the GO terms Kidney development, Spleen Development, and Bone Morphogenesis, no such defects have been observed in the 46,XY DSD patient, indicating that the currently known CBX2 variants in humans are not enough to disrupt the development of the kidney, spleen or skeletal system^5^.

Combining the previously generated DamID-Seq data with the CBX2.1 overexpression and knockdown RNA-Seq data showed that of the 1594 genes that are directly bound by CBX2.1, 228 show a significant change in expression upon CBX2.1 upregulation or ablation. Of these directly regulated genes, 116 show a negative regulation by CBX2.1 and 112 show a positive regulation by CBX2.1. Although originally described as repressors, Polycomb proteins can work as both repressors and activators as recapitulated in the review from S. Aranda *et al*.^13^.

The most interesting direct and indirect positively regulated targets of CBX2.1, concerning sex development are PDGFB, NR5A1, NR5A2, ALX4, TJP2, GH1 and DUSP6 (Figs 2 and 4). PDGFB is a growth factor that is expressed very early in the developing gonad and can form homodimers (PDGF-BB) or heterodimers with the Platelet-Derived Growth Factor Alpha (PDGFA) and has been reported to be able to produce morphological differentiation in embryonic mouse testis^14^. Additionally, it has been shown that PDGF-BB is secreted by testicular cells and induces mesonephric cell migration into the developing testis, showing its potentially vital function for testis cord formation^14,15^.

Another putative target of CBX2.1 that is expressed early on in the bipotential gonad is NR5A2. In mice, Nr5a2 has been shown to be expressed in the bipotential gonad around E11.5^16,17^. Later during mouse testis development Nr5a2 expression was observed in the testicular cords, mostly in germ cells and pre-Sertoli cells, with a declining expression pattern during testis development. Nr5a2 null mice die at E6.5 to E7.5, so its exact function during testis development has not yet been elucidated^17^. Interestingly, both NR5A1 and NR5A2 have been shown to recognize the same DNA-binding sites^16^.

NR5A1, also known as Steroidogenic Factor 1 (SF1), is an already known positively regulated target of CBX2.1^2,5,8^. SF1 is important for the regulation of gonad determination and differentiation^18^. In humans, variants in SF1 manifest with a wide spectrum of different phenotypes such as hypospadias, microphallus, infertility, undescended testis, female external genitalia and/or testicular dysgenesis in 46, XY DSD patients^19–21^.

The transcription factor ALX4 is also directly positively regulated by CBX2.1. Mice with mutations in Alx4 present with a complex phenotype, including abnormalities of the phallus and cryptorchidism^22,23^. Similar findings have been observed in human male patients with ALX4 variants and various symptoms including hypogonadism and cryptorchidism^24^.

The fifth positively regulated gene implicated in sex development is the phosphatase DUSP6. Variants in DUSP6 are implicated in Kallmann syndrome, a form of hypogonadotropic hypogonadism with delayed or absent onset of puberty and reduced testicular volume^25,26^. In granulosa cells, FSH-stimulated activation of ERK pathway appears to function through the inactivation of DUSP6, which allows for the differentiation and proliferation of the granulosa cells^27^. In the testis, DUSP6 is thought to be part of the control mechanism of cell proliferation through hormonal expression in Leydig cells^28^.

Another gene that is positively regulated by CBX2.1 is the tight junction protein TJP2, a membrane protein necessary for the assembly of tight junctions. In the testis, Sertoli cell tight junctions (SCTJs) of the seminiferous epithelium contribute to the blood-testis-barrier and are essential for spermatogenesis^29,30^. In mice, disruption of Tjp2 results in embryonic lethality and Tjp2^−/−^ chimera mice have reduced fertility and present with smaller testis with occasional degradation of the seminiferous tubules^31,32^.

GH1 is also upregulated by CBX2.1 expression, although not directly bound by it. It has been shown that failure of penile and testicular growth (peri- and postnatal) in humans can be caused by a lack of Human Growth Hormone (GH1 and GH2), despite normal androgens^33^. Intratesticular GH expression is important for the early embryonal development. GH receptors (GHR) are present in various cell types within the male reproductive system and the influence of GH on Wolffian duct differentiation has been shown in fetal rats^34–36^. Since the pituitary GH synthesis is absent or negligible during early development, this is most likely mediated by intratesticular GH production^37^. Secondly, intratesticular GH expression is important for spermatogenesis, since circulating GH from the pituitary cannot easily overcome the blood-testis barrier, so the growth hormones are mostly produced locally, which has been confirmed in human testis^38^.

Three selected genes were suppressed by CBX2.1 expression and showed an upregulation under CBX2.1 knockdown: NR2F2, TBX3, and HORMAD1. CBX2.1 directly negatively regulates the transcription factor NR2F2, a nuclear receptor that antagonizes SF1 and suppresses steroidogenesis^39^.

The two indirectly (regulated but not physically bound by) repressed targets of CBX2.1 are the transcription factor TBX3 and the chromatin binding protein HORMAD1. TBX3 is involved in developmental processes and has been shown to be expressed in the urogenital ridge of mice^40^. In humans, mutated TBX3 is associated with the ulnar-mammary syndrome, which can include genital abnormalities including micropenis, delayed puberty and cryptorchidism^41–43^.

HORMAD1 is involved in chromatin structure modulation and is mostly expressed in human testis^44^. It has been shown to be important for the meiotic prophase checkpoint in mouse and Hormad1-deficient male and female mice are infertile^45^. In humans, HORMAD1 has also been proposed to be important for male fertility, based on the finding of three SNPs found in human male patients diagnosed with infertility^46^.

In order to study the role of CBX2.1 in a stable, zero CBX2 background Sertoli-like cell line, we used CRISPR/Cas9 targeting exon1 of CBX2 in NT2-D1 cells. RNA-Seq showed that under complete CBX2 knockout, 2027 genes show a significant change in expression, compared to the CRISPR-EV control cells. To identify genes that are not only influenced by the absence of CBX2 expression, but are also bound by it, we compared the RNA-Seq with the previously conducted DamID-Seq for CBX2.1. About 10% of the genes that show a significant change in expression are predicted by the DamID-Seq to be bound by CBX2.1. Of these 214 direct targets, 167 are upregulated and 47 are downregulated, further confirming that CBX2.1 has a role as an activator of gene expression, besides its known role as a repressor. The comparison between the genes influenced under CBX2.1 transient knockdown using siRNA and the genes influenced under complete CBX2 knockout shows that there is an overlap between the two treatments of approximately 15% (Fig. 5B). The relative low overlap between siRNA and CRISPR/Cas9 could be due to the fact that in the complete CBX2 knockout, CBX2.1 has been absent for a longer period of time as well as the additive knockout of the second isoform, CBX2.2.

For further validation, CBX2.1 downstream genes were selected based on their influence on sex development and/or involvement in DSD. One of the selected genes is the transcriptional co-activator MAMLD1, a causative gene for 46,XY DSD and predicted to be directly bound by CBX2.1. MAMLD1 is downregulated in NT2-D1 cells with complete CBX2 knockout and is therefore presumably physiologically upregulated by CBX2 (Fig. 7). The core anomaly in patients with MAMLD1 variants are hypospadias, but ambiguous genitalia, cryptorchidism, micropenis and female external genitalia with complete gonadal dysgenesis have also been observed^47–49^. Like M33, Mamld1 is expressed early in gonadal development, around E11.5 in mouse Sertoli cells and E12.5 in Leydig cells^48^. In both human and mice, MAMLD1 harbors a putative SF1-binding binding sequence^47^. This implies a potential synergistic activation of MAMLD1 by CBX2.1 and SF1, which is further enforced through the previously reported upregulation of SF1 by CBX2.1.

Genes that are significantly upregulated in CBX2 KO cells, presumably downregulated *in vivo* and exclusively bound by CBX2.1 are ID4, PITX2, ERBB2, and NTF3 (Fig. 7).

ID4, also known as Inhibitor of Differentiation 4, is highly expressed in Sertoli cells and the expression of Id4 start between E7.5 and E9.5^50,51^. The exact role of ID4 during testis development has not been elucidated. However, Id4 is also expressed in granulosa cells of XX mice and Id4 deficiency leads to diminished estrogen levels^52^.

The transcription factor PITX2 is also directly bound and upregulated in CBX2 KO cells. In chicken gonads, Pitx2 mRNA is only observed in the left gonad, which develops into a functional ovary, and not the right^53^. Additional significance for Pitx2 in gonad development was found in rat gonads, where Pitx2 is expressed equally in XY and XX gonads at E14.5 in the bipotential gonad and then the expression diminishes in male gonads, while the expression is maintained in female gonads^54^. Basu M., *et al*. found that PITX2 activates the Wnt signaling pathway and interacts with the promoter regions of WNT family members in ovarian cancer cells (SKOV3). Furthermore, overexpression induces upregulation of β-catenin^55^.

ERBB4 is an epidermal growth factor highly expressed in testis and plays a role in testis development and in fertility^56,57^. In mice, its expression pattern is sexually dimorphic and it has been proposed to function as an advancement factor for testis development by coordinating communication between Sertoli, Leydig and germ cells. This hypothesis is supported by the compromised 3D organization of the seminiferous tubules in Erbb4 knockout mice^56^.

Another target of CBX2.1 that is upregulated in CBX2 KO cells is Neurotrophin 3 (NTF3). It has been shown that NTF3 is a direct target of SRY and SOX9, and is produced in Sertoli cells as a chemoattractant for myoid cells, which is an important process for the formation of the seminiferous tubules^58,59^. However, mice lacking Ntf3 still develop normal testis, which implies the presence of a redundant mechanism^60^. The expression of neurotrophinsis also known to be important for ovarian development and NTF3 and its receptor trkC are expressed in the rat ovary already at day 18 of fetal development^61,62^. CBX2.1 potentially acts as a control mechanism for the regulated chemoattraction of myoid cells, helping to coordinate the timeline of proper testis development. Additionally, ablation of Sertoli cells in mice leads to a rapid dedifferentiation of peritubular myoid cells, indicating a paracrine effect, in which Ntf3 could play a part^63^.

**Figure 8:**
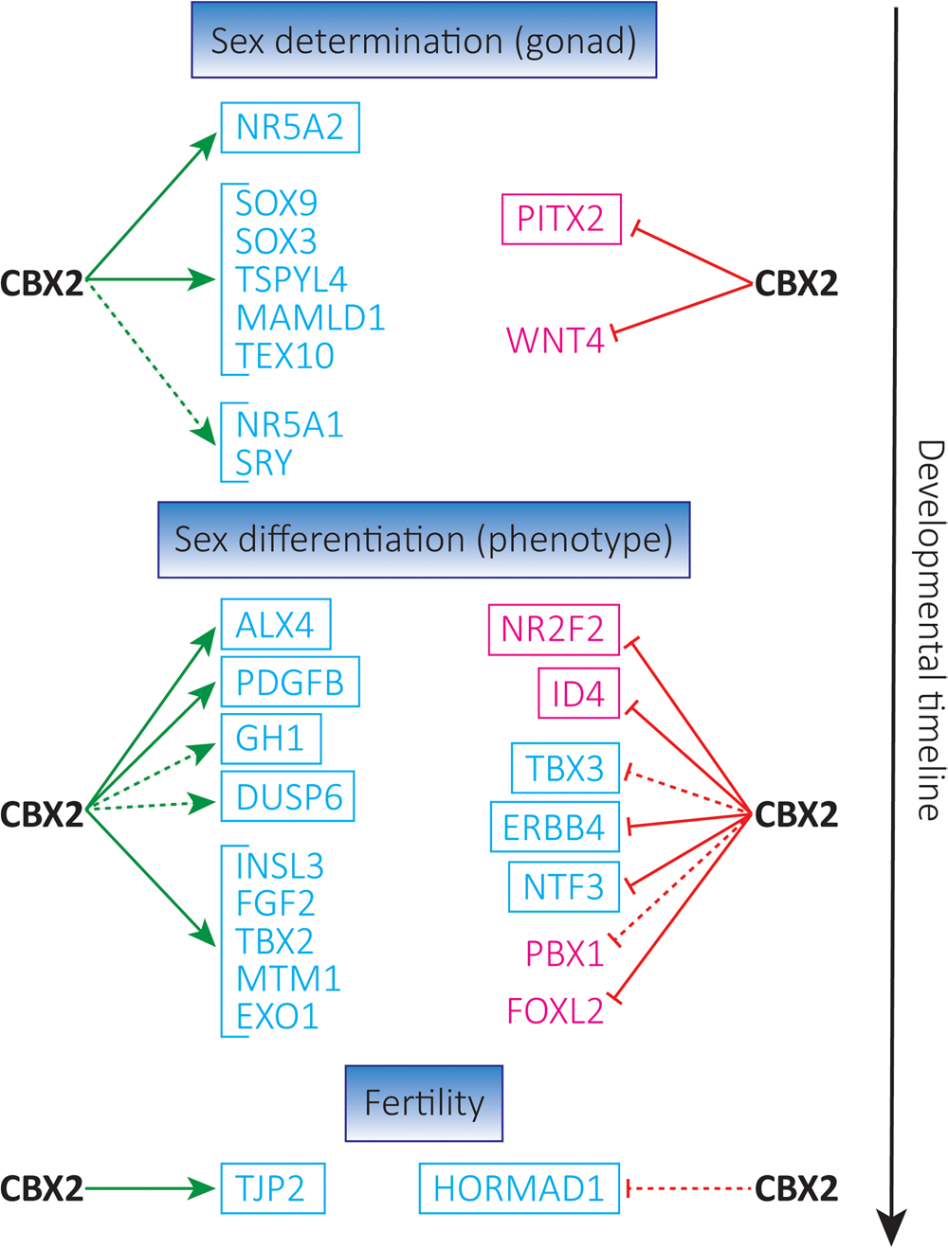
Influence of CBX2.1 on its direct and indirect targets. The targets were divided between Sex determination, Sex differentiation and Fertility. In blue the male and in pink the female factors. Positive regulation by CBX2.1 is depicted in green, negative regulation in red. Broken lines indicate an indirect interaction of CBX2.1 with the downstream gene. The genes that were newly added as downstream targets of CBX2.1 are depicted in boxes, while targets previously identified using DamID-Seq and qRT-PCR by Eid *et al*. are without boxes^7^. NR5A1 and MAMLD1 were part of the identified targets in both studies.

The clinical importance of CBX2.1 was highlighted by the diagnosis of CBX2 deficiency in 46,XY patients^5,9^. Here, we expand on the previously gained insight by Eid *et al*. on the downstream targets of CBX2.1^8^. While Eid *et al*. selected genes based on the proposed binding of CBX2.1 near translation start sites (TSS) of downstream targets using DamID-Seq, we analyzed the CBX2.1 transcriptome by looking at target genes significantly regulated under different CBX2.1 expression conditions (Fig. 8). That some of the genes showing a significant regulation when analyzed with RNA-Seq were not significantly regulated according to RT-qPCR, and *vice versa*, might be ascribed to the differences in the two methods with different biases and limitations.

In this study, we confirmed the crucial role of CBX2.1 in the regulation of renowned factors such as SF1 and added new candidates for sex development and its defects, e.g. NR5A2. Additionally, it appears that CBX2.1 influences directly testis determination in its early stages, via regulation of SF1, NR5A2, PITX2 and MAMLD1, as well as sex differentiation by regulating the cross-talk between Sertoli, Leydig and germ cells (via ERBB4) and steroidogenesis, by regulation of NR5A1 and NR2F2. Generally speaking, gonadal determination is a balance between male and female factors. As such, CBX2.1 seems to promote the male pathway and actively inhibit the female. Eid *et al*. have previously shown this, discovering that CBX2.1 directly inhibits WNT4 and PBX1 and indirectly inhibits the expression of FOXL2^8^. Here we add PITX2 and ID4 to the list of female factors that are directly inhibited by CBX2.1, further implicating CBX2.1 as a pro male factor. However, CBX2.1 appears to be important not only for testis development but also for the maintenance of proper testicular function postnatal, as suggested by the regulation of GH1. We also gather hints that CBX2.1 is potentially implicated in spermatogenesis and male fertility through the downstream targets TJP2 and HORMAD1. This is an important venue of investigation, not only to advance our understanding of one of the most fundamental processes in most multicellular life forms, but also to expand our ability to diagnose, counsel and properly accompany DSD patients and their families.

## Methods

### Cell culture

NTERA-2 cl. D1 (NT2-D1, ATCC CRL-1973) cells were cultured at 37 °C and 10% CO_2_ in Dulbecco’s Modified Eagle’s Medium (D5796, Sigma-Aldrich, St. Louis MO, United States) containing 10% fetal calf serum (2-01F310-I, BioConcept Ltd. Amimed) and supplemented with 5% penicillin/streptomycin (10′000 U/ml penicillin, 10′000 µg/mL streptomycin, 15140122 Gibco, ThermoFisher Scientific).

### Overexpression

Complementary DNA of CBX2.1 (NM_005189) in a pCMV6-XL5 vector was purchased from Origene (Rockville, Maryland, United States). 4 µl/µg DNA of Fugene 6 (Promega, Madison, Wisconsin, United States) was used for the transfection of NT2-D1 cells with either CBX2.1 or an EV as a control. The total RNA was extracted 48 h after transfection, using the RNA extraction kit from Qiagen (Hilden, Germany) according to the manufacturer instructions.

### siRNA

siRNA duplexes were purchased from Microsynth (Balgach, Switzerland). The sequences of the siRNA used are the following: siScrambled (5′-CGUACGCGGAAUACUUCGATT-3′)^6^, CBX2-145 (5′-GGCUGGUCCUCCAAACAUATT-3′) and CBX2-411 (5′-GGAUGACAGUGAGUUAGAUTT-3′). LipofectamineRNAiMAX (Invitrogen, Carlsbad CA, United States) was used as transfection reagent, siRNA duplexes were transfected at a final concentration of 40 nM. Again, total RNA was extracted after 48 h using the RNA extraction kit from Qiagen.

### CRISPR/Cas9

NT2-D1 cells with complete CBX2 knockout were created, according to the instructions of Ran F.A. *et al*. and using the empty CRISPR-Cas9 construct with puromycin cassette (pSpCas9(BB)-2A-Puro (PX459) V2.0 (Addgene plasmid #62988)) from Origene. Briefly, 20 bp guide RNA specific for CBX2 was created using the CRISPR Design Tool (http://tools.genome-engineering.org), two complementary ssRNAs were ordered from Microsynth (Switzerland) and subsequently phospholylated and annealed. These oligos were then cloned into the CRISPR-Cas9 vector. Finally, the vector was transformed and amplified in DH5α competent *E.Coli* cells, extracted using theQIAprep Spin Miniprep Kit (Qiagen) according to the manufacturer’s manual and sequenced on a 3500 Genetic Analyzer (Applied Biosystems). NT2-D1 cells were either transfected with the CBX2 targeting CRISPR-Cas9 construct or a control CRISPR-Cas9 construct with a scrambled guide RNA. The cells were cultured in the previously mentioned DMEM medium for two weeks, complemented with 0.5 µg/ml of puromycin for conditional selection. The CBX2 knockout was confirmed through western blot and immunofluorescence (Supplemental Fig. 2A,B).

### RNA-sequencing

Total RNA samples from Sertoli-like cell (NT2-D1) triplicates, which have been transfected with either WT CBX2.1, empty vector (EV), siRNA against CBX2.1 or scrambled siRNA, were analysed by RNA-Seq on a HiSeq. 2500 Sequencer (Illumina, San Diego CA, United States), as well as triplicates of CBX2-KO and NT2-D1 CRISPR control RNA samples. The reads were screened with FastQ Screen (Babraham Bioinformatics) for possible contamination and a quality control has been performed with FastQC (Babraham Bioinformatics). RSEM (Dewey Lab) was used to quantify the gene expression level and the differential expression between samples, including the corresponding false discovery rate (FDR), was calculated by EdgeR (Bioconductor). The FDR is defined as the probability of a false-positive discovery, taking into account the total number of null hypotheses tested over the whole experiment. All differential expressions with a FDR below 0.05 were defined as significant. STAR (Spliced Transcripts Alignment to a Reference) was used to map the RNA reads to the reference sequence.

### Genome-wide analysis of CBX2 binding sites

In order to gain insight into the CBX2 protein/DNA interaction, the DamID (DNA adenine methyltransferase identification) assay coupled with Next Generation Sequencing (NGS) was used as previously described^8^.

### Gene-ontology (GO) enrichment analysis

ToppCluster was used for GO-enrichment analysis of CBX2.1 target genes. GO-enrichment allows for the analyzation of functional features of gene sets, clustering them by their involvement in pathways related to Molecular Function, Biological Process and/or Cellular Component. GO-terms with p-values ≤ 0.05 and more than three target genes associated to the corresponding GO-term were defined as significant. CBX2.1target genes (e.g. after CBX2.1 knockdown or overexpression, respectively CBX2-KO vs NT2-D1 control) were clustered depending on GO-terms and visualized using spring-embed layout with Cytoscape v3.7.1. The GO terms involved in Biological Process were split into subcategories (Developmental Process, Regulatory Process etc.), while Molecular Function is shown as a whole.

### Quantitative real-time PCR

Extracted total RNA was reverse-transcribed using Omniscriptreverse-transcriptase (Qiagen, Hilden, Germany) according to the manufacturer instructions. All experiments were performed on an ABI StepOnePlus Real-Time PCR (Thermo Fisher Scientific, Waltham MA, United States) and the PCR products were quantified fluorometrically using the KAPA SYBR FAST master mix (Roche, Basel, Switzerland). To normalize the data, the mRNA level of cyclophilin was used (primer sequences available upon request). All samples were run at least in triplicates, unpaired t-test was performed using GraphPad Prism (v.6.0.7, GraphPad Software, La Jolla CA,United States) and the data are given as mean ± SEM (Standard Error of the Mean).

### Western blot

Proteins for western blot were either harvested from cells lysed with NP40 Lysis Buffer (J619-500ML, Amrescor) supplemented with 0.1% PMSF Protease Inhibitor (36978, ThermoFisher Scientific, Waltham MA, United States) or during RNA-extraction according to the manual “Acetone Precipitation of Protein from Buffer RLT Lysates” (RNeasy Mini Handbook, Qiagen, Germany) during RNA extraction. CBX2 protein levels were visualized with an ImageQuant LAS 4000 (GE Healthcare, Life Sciences), using chemilumenescence. As a loading control, β-tubulin protein levels were analyzed.

### Immunofluorescence

CBX2-KO cells transfected with CBX2 or an empty vector, were compared to NT2-D1 CRISPR control cells, regarding CBX2 expression. Cells were grown on sterilized coverslips in 6-well plates. After 48 h of transfection, the cells were fixated with 4% Formaldehyde in PBS for 15 min. The cells were washed with PBS and permeabilized with 0.2% TritonX in PBS for 5 min. The cells were washed with PBS and then blocked with 3% milk in PBS for 30 min. The cells were incubated with the primary antibody (anti-CBX2, Rabbit, 1:100) in 3% milk for 2 h. After washing with PBS, the cells were incubated with the second antibody (Alexa Fluor 488 anti-rabbit) in 3% milk for 1 h and then washed again for 15 min with PBS. Mounting media (Vectrashield with DAPI, Vector Laboratories) was used and the Immunofluorescence was visually analyzed using an Olympus CKX41 Microscope.

## Supplementary information

Supplementary Information

## Data Availability

All data generated and/or analyzed during the current study are available from the corresponding author on reasonable request.

## References

[1] Lanzuolo C, Orlando V (2012). Memories from the Polycomb Group Proteins. Annu. Rev. Genet..

[2] Katoh-Fukui Y (1998). Male-to-female sex reversal in M33 mutant mice. Nature..

[3] Katoh-Fukui Y (2006). Mouse Polycomb M33 is required for splenic vascular and adrenal gland formation through regulating Ad4BP/SF1 expression. Blood..

[4] Katoh-Fukui Y (2012). Cbx2, a polycomb group gene, is required for Sry gene expression in mice. Endocrinology..

[5] Biason-Lauber A, Konrad D, Meyer M, deBeaufort C, Schoenle EJ (2009). Ovaries and Female Pheotype in a Girl with 46,XY Karyotype and Mutations in the CBX2 Gene. Am. J. Hum. Genet..

[6] Ostrer H, Huang HY, Masch RJ, Shapire EA (2007). Cellular Study of Human Testis Development. Sex. Dev..

[7] Li, L. *et al*. Single-Cell RNA-Seq Analysis Maps Development of Human Germline Cells and Gonadal Niche Interactions. *Cell Stem Cell* **20**, 858–873, *GEO accession:* **GSE86146** (2017). 10.1016/j.stem.2017.03.007 28457750

[8] Eid W, Opitz L, Biason-Lauber A (2015). Genome-wide identification of CBX2 targets: insights in the human sex development network. Mol. Endocrinol..

[9] Sproll P (2018). Assembling the jigsaw puzzle: CBX2 isoform 2 and its targets in disorders/differecens of sex development. Mol. Genet. Genomic. Med..

[10] R Development Core Team. R: A language and environment for statistical computing. *R Foundation for Statistical Computing*. ISBN 3-900051-07-0, http://www.R-project.org (2008).

[11] Warnes, G. R. *et al*. gplots: Various R Programming Tools for Plotting Data, https://CRAN.R-project.org/package=gplots (2016).

[12] Bouazzi L, Franco MA, Maret A, Biason-Lauber A (2018). CBX2 and the Ovary: Novel Insights into Regulatory Networks in Humans. J. Gynecol. & Reprod. Med..

[13] Aranda, S., Mas, G. & Di Croce, L. Regulation of gene transcription by Polycomb proteins. *Sci. Adv*. **1**10.1126/sciadv.1500737 (2015). 10.1126/sciadv.1500737 PMC4672759 26665172

[14] Ricci G, Catizone A, Galdieri M (2004). Embryonic mouse testis development: role of platelet derived growth factor (PDGF-BB). J. Cell. Physiol..

[15] Puglianiello A (2004). Expression and role of PDGF-BB and PDGFR-β during testis morphogenesis in the mouse embryo. J. Cell. Sci..

[16] Volle DH (2006). The small heterodimer partner is a gonadal gatekeeper of sexual maturation in male mice. Genes. Dev..

[17] Hinshelwood MM, Shelton JM, Richardson JA, Mendelson CR (2005). Temporal and Spatial Expression of Liver Receptor Homologue-1 (LRH-1) During Embryogenesis Suggests a Potential Role in Gonadal Development. Dev. Dyn..

[18] Biason-Lauber A (2010). Control of sex development. Best. Pract. Res. Clin. Endocrinol. Metab..

[19] Tuhan, H., *et al*. A novel mutation in steroidogenic factor (SF1/NR5A1) gene in a patient with 46,XY DSD without adrenal insufficiency. *Andrologia*. **49**, 10.1111/and.12589 (2017). 10.1111/and.12589 27135758

[20] Nishina-Uchida N (2013). Characterisitc Testicular Histology is Useful for the Identification of NR5A1 Gene Mutations in Prepubertal 46,XY Patients. Horm. Res. Paediatr..

[21] Mallet D (2004). Gonadal Dysgenesis Without Adrenal Insufficiency in a 46, XY Patient Heterozygous for the Nonsense C16X Mutation: A Case of SF1 Happloinsufficiency. J. Clin. Endocrinol. Metab..

[22] Meijlink F, Beverdam A, Brouwer A, Oosterveen TC, Berge DT (1999). Vertebrate aristaless-related genes. Int. J. Dev. Biol..

[23] Matsumaru D (2014). Genetic analysis of the role of Alx4 in the coordination of lower body and external genitalia formation. Eur. J. Hum. Genet..

[24] Kayserili H (2009). ALX4 dysfunction disrupts craniofacial and epidermal development. Hum. Mol. Genet..

[25] Valdes-Socin H (2014). Reproduction, smell and neurodevelopmental disorders: genetic defects in different hypogonadotropic hypogonadal syndromes. Front. Endocrinol..

[26] Miraoui H (2013). Mutations in FGF17, IL17RD, DUSP6, SPRY4, and FLRT3 Are Identified in Individuals with Congenital Hypogonadotropic Hypogonadism. Am. J. Hum. Genet..

[27] Donaubauer EM, Law NC, Hunzicker-Dunn ME (2016). Follicle-Stimulating Hormone (FSH)-dependent Regulation of Extracellular Regulated Kinase (ERK) Phosphorylation by the Mitogen-activated Protein (MAP) kinase Phosphatase MKP3. J. Biol. Chem..

[28] Mori Sequeiros Garcia M (2013). MAP kinase phosphatase-3 (MKP-3) is transcriptionally and post-translationally up-regulated by hCG and modulates cAMP-induced p21 expression in MA-10 Leydig cells. Mol. Cell. Endocrinol..

[29] Chakraborty P (2014). Androgen-dependent sertoli cell tight junction remodeling is mediated by multiple tight junction components. Mol. Endocrinol..

[30] Gruber M, Mathew LK, Runge AC, Garcia JA, Simon MC (2010). EPAS1 is Required for Spermatogenesis in the Postnatal Mouse. Biol. Reprod..

[31] Fanning AS, Anderson JM (2009). Zonula occludens-1 and -2 are cytosolic scaffolds that regulated the assembly of cellular junctions. Ann. N. Y. Acad. Sci..

[32] Xu J (2009). Zona occludens-2 is critical for blood-testis barrier integrity and male fertility. Mol. Biol. Cell..

[33] Laron Z, Sarel R (1970). Penis and testicular size in patients with growth hormone insufficiency. Acta. Endocrinol..

[34] García-Aragón J (1992). Prenatal expression of the growth hormone (GH) receptor/binding protein in the rat: a role for GH in embryonic and fetal development?. Development..

[35] Lobie PE, Breipohl W, Aragón JG, Waters MJ (1990). Cellular localization of the growth hormone receptor/binding protein in the male and female reproductive systems. Endocrinology..

[36] Nguyen AP, Chandorkar A, Gupta C (1996). The role of growth hormone in fetal mouse reproductive tract differentiation. Endocrinology..

[37] Sanders EJ, Harvey S (2004). Growth hormone as an early embryonic growth and differentiation factor. Anat. Embryol..

[38] Hull KL, Harvey S (2000). Growth hormone: roles in male reproduction. Endocrine..

[39] Litchfield LM, Klinge CM (2012). Multiple roles of COUP-TFII in cancer initiation and progression. J. Mol. Endocrinol..

[40] Douglas NC, Heng K, Sauer MV, Papaioannou VE (2012). Dynamic Expression of Tbx2 Subfamily Genes in Development of the Mouse Reproductive System. Dev. Dyn..

[41] Tanteles GA (2017). Novel TBX3 mutation in a family of Cypriot ancestry with ulnar-mammary syndrome. Clin. Dysmorphol..

[42] Sasaki G (2002). Novel Mutation of TBX3 in a Japanese Family With Ulnar-Mammary Syndrome: Implication for Impaired Sex Development. Am. J. Med. Genet..

[43] Linden H, Williams R, King J, Blair E, Kini U (2009). Ulnar Mammary Syndrome and TBX3: Expanding the Phenotype. Am. J. Med. Genet..

[44] Chen YT (2005). Identification of CT46/HORMAD1, an immunogenic cancer/testis antigen encoding a putative meiosis-related protein. Cancer. Immun..

[45] Kogo H (2012). HORMAD1-dependent checkpoint/surveillance mechanism eliminates asynaptic oocytes. Genes. Cells..

[46] Miyamoto T (2012). Singel-nucleotide polymorphisms in HORMAD1 may be a risk factor for azoospermia caused by meiotic arrest in Japanese patients. Asian. J. Androl..

[47] Ogata T, Laporte J, Fukami M (2009). MAMLD1 (CXorf6): A New Gene Involved in Hypospadias. Horm. Res..

[48] Ruiz-Arana IL (2015). A Novel Hemizygous Mutation of MAMLD1 in a Patient with 46,XY Complete Gonadal Dysgenesis. Sex. Dev..

[49] Ogata T, Sano S, Nagata E, Kato F, Fukami M (2012). MAMLD1 and 46,XY Disorders of Sex Development. Semin. Reprod. Med..

[50] Chaudhary J, Johnson J, Kim G, Skinner MK (2001). Hormonal regulation and differential actions of the helix-loop-helix transcriptional inhibitors of differentiation (Id1, Id2, Id3, and Id4) in Sertoli cells. Endocrinology..

[51] van Crüchten I (1998). Structure, chromosomal localization and expression of the murine dominant negative helix-loop-helix Id4 gene. Biochim, Biophys. Acta..

[52] Best SA (2014). Dual roles for Id4 in the regulation of estrogen signaling in the mammary gland and ovary. Development..

[53] Rordiguez-León J (2008). Pitx2 regulates gonad morphogenesis. PNAS..

[54] Nandi SS, Ghosh P, Roy SS (2011). Expression of PITX2 homeodomain transcription factor during rat gonadal development in a sexually dimorphic manner. Cell. Physiol. Biochem..

[55] Basu M (2012). Wnt/β-catenin pathway is regulated by PITX2 homeodomain protein and thus contributes to the proliferation of human ovarian adenocarcinome cell, SKOV-3. J. Biol. Chem..

[56] Naillat F (2014). ErbB4, a Receptor Tyrosine Kinase, Coordinates Organization of the Seminiferous Tubules in the Developing Testis. Mol. Endocrinol..

[57] Chen SU, Liu YX (2015). Regulation of spermatogonial stem cell self-renewal and spermatocyte meiosis by Sertoli cell signaling. Reproduction..

[58] Cupp AS, Uzumcu M, Skinner MK (2003). Chemotactic Role of Neurotropin 3 in the Embryonic Testis That Facilitates Male Sex Determination. Biol. Reprod..

[59] Clement TM, Bhandari RK, Sadler-Riggleman I, Skinner MK (2011). SRY Directly Regulates the Neurotrophin 3 Promoter During Male Sex Determination and Testis Development in Rats. Biol. Reprod..

[60] Levine E, Cupp AS, Skinner MK (2000). Role of Neurotropins in Rat Embryonic Testis Morphogenesis (Cord Formation). Biol. Reprod..

[61] Ojeda SR, Romero C, Tapia V, Dissen GA (2000). Neurotrophic and cell-cell dependent control of early follicular development. Mol. Cell. Endocrinol..

[62] Dissen GA, Hirshfield AN, Malamed S, Odjeda SR (1995). Expression of neurotrophins and their receptors in the mammalian ovary is developmentally regulated: changes at the time of folliculogenesis. Endocrinol..

[63] Rebourcet D (2014). Sertoli cells control peritubular myoid cell fate and support adult Leydig cell development in the prepubertal testis. Development..

