# Supplementary material for: CBX2-dependent transcriptional landscape: implications for human sex development and its defects
Source: Sci Rep. 2019 Nov 12;9:16552. doi: 10.1038/s41598-019-53006-7 (PMC6851130; doi:10.1038/s41598-019-53006-7)
Supplement: Supplementary file 1 — Supplementary Information [file 41598_2019_53006_MOESM1_ESM.pdf]

CBX2-dependent transcriptional landscape: implications for human sex development and its defects

Patrick Sproll[1], Wassim Eid[1,2], Anna Biason-Lauber[1*]

[1]Division of Endocrinology, Dept. of Medicine, University of Fribourg, Fribourg, 1700, Switzerland

[2]Department of Biochemistry, Medical Research Institute, University of Alexandria, Alexandria, 21526, Egypt

Supplementary Information

S1: The Heatmap was created with R (BioconductR) to show the significant differently expressed genes between CBX2.1 treatments and to visualize the similarity between treatments. The color key is given as a Row Z-score (centered and normalized expression as x standard deviations from the mean). si: cells treated with siRNA against CBX2.1; Scr: cells treated with scrambled siRNA (siRNA control); Over: cells transfected with CBX2.1; EV: cells transfected with an empty vector (overexpression control). A through C are the triplicate repetitions.

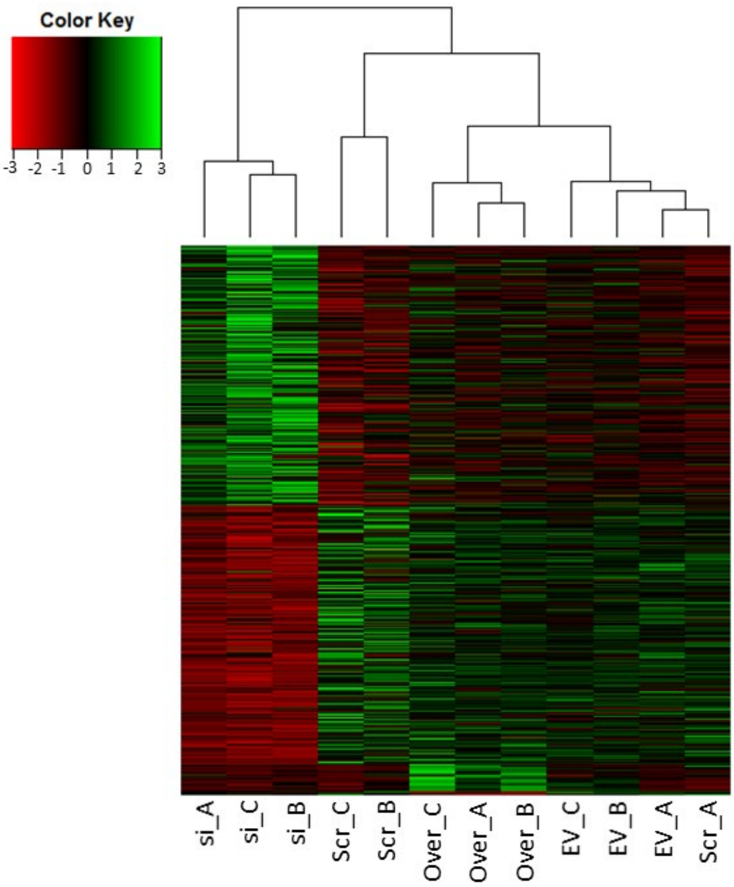

S2: ***A.*** Confirmation western blot for absence of CBX2.1 expression in CBX2 CRISPR/Cas9 NT2-D1 cells with all the appropriate controls (CBX2.1 overexpression, siRNA against CBX2.1 and EV CRISPR/Cas9). ***B.*** Immunofluorescence of NT2-D1 cells transfected with CRISPR/Cas9 against CBX2 and cells transfected with EV CRISPR/Cas9.

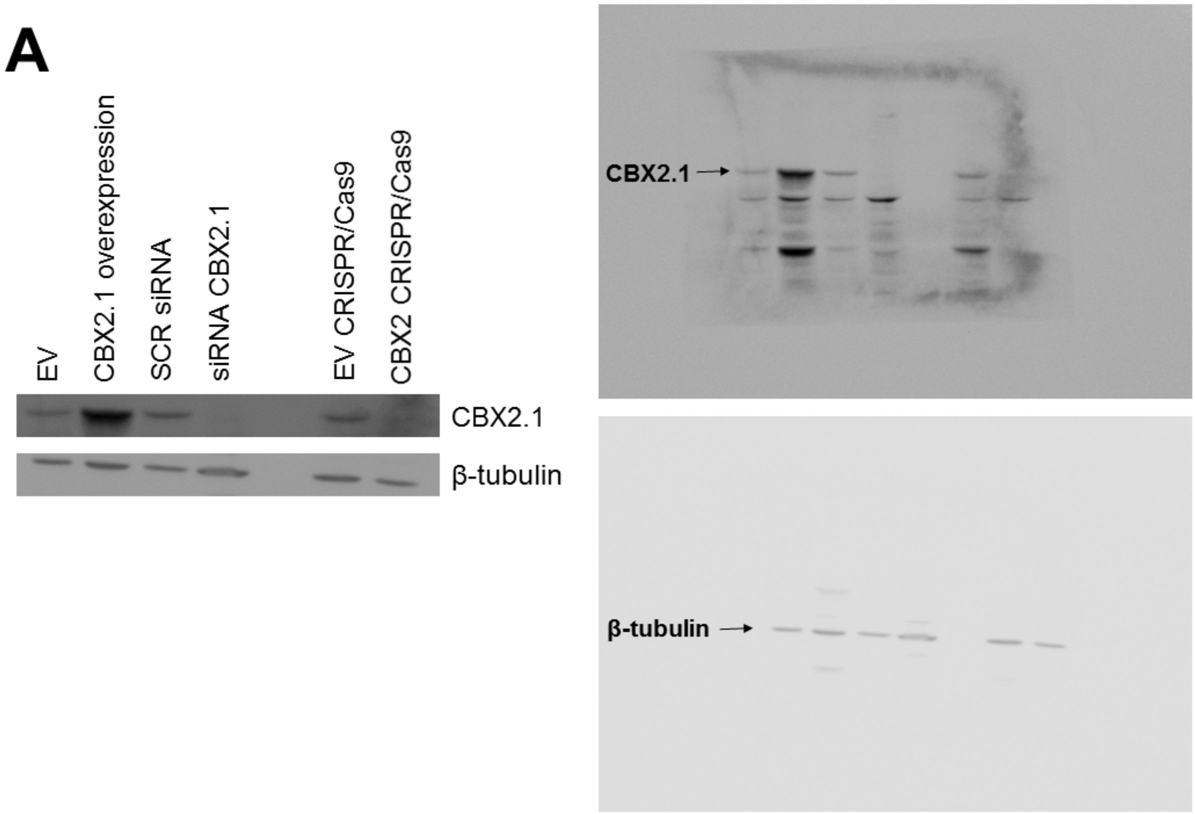

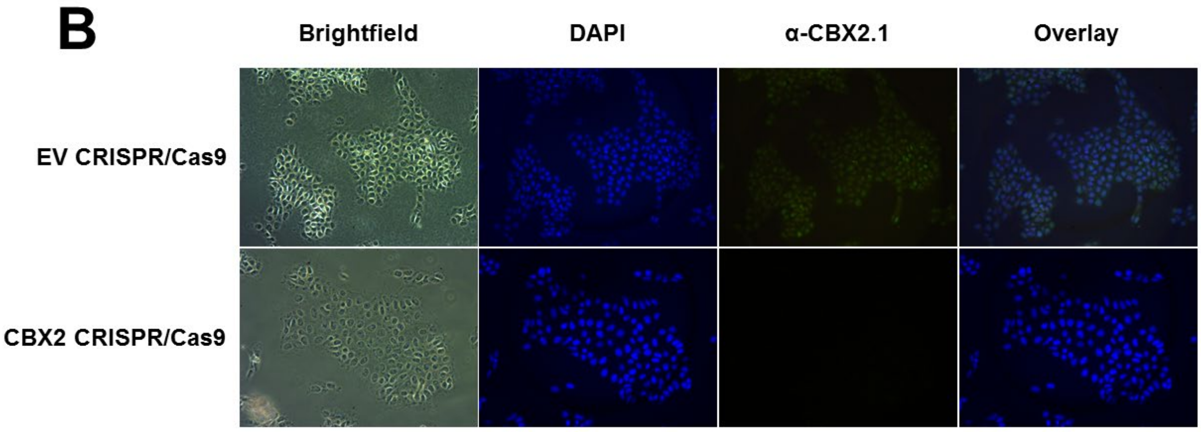

S3: Analysis of the RNA-Seq data with the help of the IGV viewer.
CRISPR/Cas9 treatment of NT2-D1 cells led to the insertion of an Adenine (c.44insA), which led to a frameshift mutation and a premature stop codon (p.Glu15Glufs25Ter), which is not present in the EV.

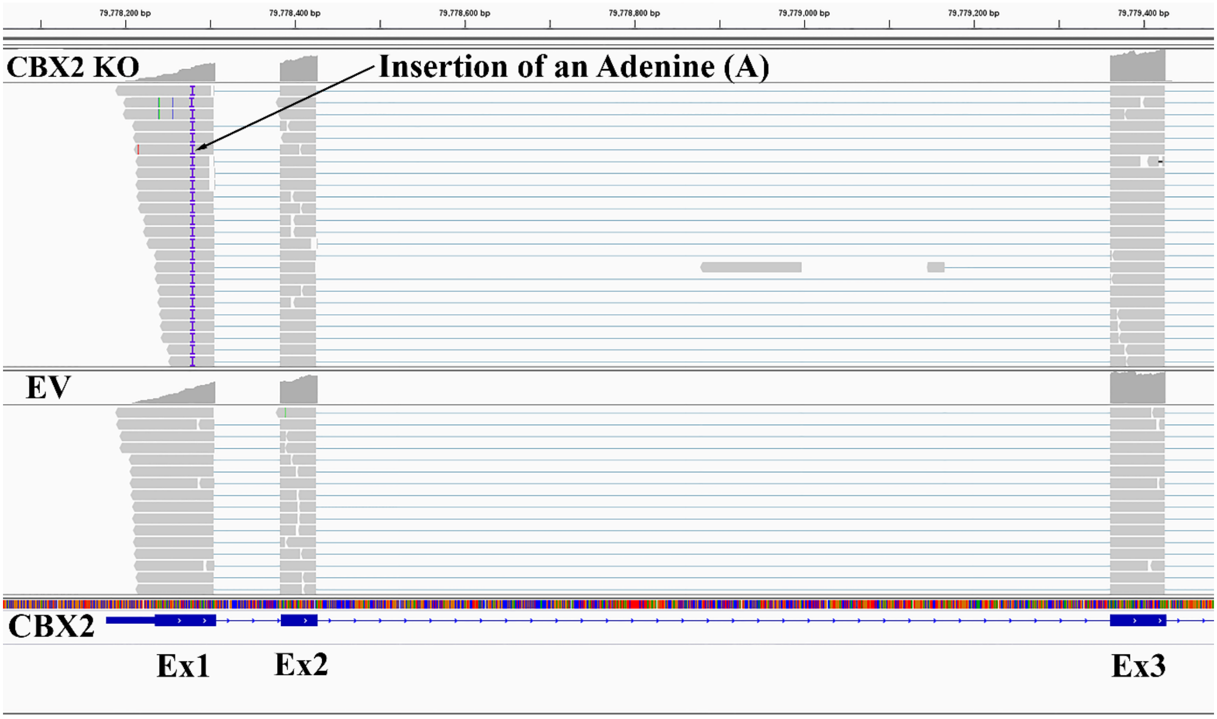

S4: Supplemental table for Fig. 3 "Venn diagram comparing gene regulation".

**A. Genes common between CBX2.1 knockdown and CBX2.1 overexpression**								
ACTA2	CALD1	EPPK1	GAL3ST4	ITGAV	MYOF	RPS27L	TIAF1	VGF
AHNAK2	CNTN2	EPS8L2	GDF15	KIF1A	PHLDA1	SESN1	TIMP3	
ANO4	CSNK1G1	FAM212B	GRHL3	LOX	PHLDA3	SLC5A3	TP53I3	
AP001468.1	CYFIP2	FAS	HSPB8	LY6K	PLK2	SLFN13	TP53INP1	
BTG2	DRAM1	FOSL1	IGF2	MAGED4	RNF144B	SP140	TSPAN11	
C12orf5	EPAS1	FRMD6	INPP5D	MFAP5	RP11-438J1.1	SULF2	UNG	
**B. Genes regulated under CBX2.1 overexpression and bound by CBX2.1 (DamID)**								
KCNJ2	PALLD	TGFB2	TNFRSF10D					
**C. Genes regulated under CBX2.1 knockdown and bound by CBX2.1 (DamID)**								
ABHD5	CBLB	DIS3L2	GPC6	KLHL13	NR5A2	PTBP2	SLC25A24	TPM2
ABL2	CCND2	DNAJC10	GPM6A	LDB2	NTNG1	PTN	SLC40A1	TRIM9
ACSL4	CCNG2	DPYSL3	GREB1L	LHX8	NTPCR	PTPLAD1	SLC4A3	UBE2Q2
ADAMTS9	CD164	DPYSL5	GRID2	LRGUK	OSBPL8	PTPLB	SLITRK1	UNC80
AEBP2	CDC27	DSCAM	GXYLT1	MAN1A1	OTUD4	PTPRG	SMC5	USP33
AKAP13	CDCA7	DSE	HDDC2	MAP3K15	PBX1	RABGAP1L	SOX4	USP6NL
AKAP7	CHD7	DTL	HECA	MAP3K5	PBX3	RAD51AP1	SOX5	UST
ALG13	CHN1	EBPL	HMCN1	MARK2	PCLO	RAD51B	SP8	VBP1
ALX4	CHRNB2	EDN1	HNRNPA1L2	MBNL3	PCSK9	RAP2A	SSPN	VEPH1
AMOT	CKB	ELK3	IDS	MCCC2	PDE10A	RASGEF1B	SYNDIG1	WDR11
ANO2	CNKSR2	EML1	IKZF2	MDFIC	PDE3A	RBBP7	SYT1	XPNPEP1
APPL1	CNOT8	EPDR1	INTS6	MECOM	PDE4B	RBM26	TANC1	YWHAB
ARFGAP2	COBL	ERC1	IRS1	MEIS1	PDGFB	RGMA	TDRKH	ZFHX4
ARHGAP12	COL13A1	ERRFI1	ITGA9	MFAP3	PDZD8	RGS2	TEK	ZNF280C
ARHGAP5	CTNND1	EZH2	ITPR2	MFSD8	PHF14	RLF	TERT	ZNF608

ARNT2	CUL5	FAM129A	JAG1	MID1	PKN2	RND3	TFDP2	ZNF618
ARNTL	CXorf57	FAM160B1	KANK4	MPP6	PKP2	ROBO1	THSD7B	ZNF664
ASXL3	CYTIP	FEZF1	KATNA1	MTMR7	PLD5	ROR1	TJP2	ZNF708
ATG4C	DAB1	FNBP1L	KCND2	MUM1L1	PLEKHA2	SCAPER	TLE4	ZRANB2
ATP6V1B1	DACT3	GALNT2	KCNH8	MYB	PLXNA2	SCD	TMEM132B	
ATRNL1	DARS2	GBX2	KCTD12	MYO10	POU2F1	SEMA3D	TMEM2	
BBS9	DCBLD1	GFRA1	KDM5B	MZT1	PPP1CB	SESTD1	TMEM50B	
BTG3	DDAH1	GJA1	KIAA1919	NFIA	PRKX	SH3BP4	TMOD1	
CABYR	DEPDC1	GNA12	KIF26B	NFYB	PRPF38B	SH3GLB1	TNFSF4	
CAND1	DIS3	GOLIM4	KLF5	NR2F2	PSMD10	SKAP2	TNIK	
**D. Genes bound by CBX2.1 (DamID) and influenced by both CBX2.1 knockdown and overexpression**								
ANK1	CBX2	CDKN1A	GADD45A	TNFRSF10C				

S5: Supplemental table for Fig. 5A “NGS target comparison”. All 214 genes significantly regulated under complete CBX2 knockout and bound by CBX2.1 (DamID)

ACADL	BOC	DLEU7	FZD4	IRX3	MECOM	PCDH9	SCN3A	TMC1
ADAMTSL3	C1orf21	DNER	FZD7	ITGA11	MEIS1	PCSK9	SEMA3C	TMEM132B
ADCY10	C8orf4	DSCAM	GADD45A	ITGA9	MID1	PDCD4	SERPINI2	TMEM132C
ADRB2	C8orf48	EBF2	GATA3	KANK4	MMP19	PDE3A	SGIP1	TMEM217
AGT	CA8	EDN1	GBX2	KAT2B	MOXD1	PDE4B	SIM1	TMOD1
AKAP6	CAPN6	EDNRA	GNA14	KCND2	MPP6	PDGFB	SKAP2	TNFSF12
AKAP7	CCDC80	EFNA5	GPC3	KCNH1	MUM1L1	PITX2	SLC30A4	TNFSF4
ALDH1A2	CCK	EML1	GPM6A	KCNMA1	MYL9	PKP2	SLC40A1	TNIK
ALX4	CCNG2	ENTPD1	GREB1L	KIAA0226L	NCKAP5	PLA2G4A	SLC8A1	TNNI1
AMOT	CDKN2B	EPB41L2	GRIA3	KIF26B	NEGR1	PLB1	SMAD6	TRDN
ANO2	CHST4	ERBB4	GRID2	LAMA2	NKX6-1	PLXNA2	SMOC2	TTLL7
AOAH	CLDN2	ESAM	GRM3	LAMA4	NOL4	PM20D2	SOX5	UNC5C
ARHGAP10	CNTN3	ESRRG	HIST3H2A	LDB2	NOTCH2	PRICKLE2	SOX6	VAMP8
ARHGAP15	COL11A1	ETV1	HPSE2	LGR6	NPAS3	PROX1	SOX9	VAV3
ARHGAP20	COL13A1	EVX1	HS3ST5	LHX5	NR2F2	PRRX1	SPAG17	VEPH1
ARHGAP24	COL2A1	EYA4	HTR2C	LHX8	NR3C2	RASGRF1	SPRY3	VIT
ARHGAP36	CRB1	FAM65B	HYDIN	LMX1A	NR5A2	RBFOX1	STMN4	VWF
ARHGEF17	CSRNP3	FEZF1	ID4	LRRN3	NTF3	RNF182	SYTL3	WNT8B
ARHGEF6	CTGF	FGGY	IDS	LTBP2	NTNG1	RPE65	TBX5	ZFHX4
ARMC3	DACH1	FHIT	IDS	MAMLD1	OTOGL	RSPH4A	TEK	ZNF385C
ASXL3	DACT1	FOLR1	IGFBP7	MAP1LC3C	PALLD	RSPO3	TEX13B	ZNF536
ATP6V1B1	DACT3	FOXC1	IKZF2	MAP2	PAPPA2	RXRG	TGFB2	ZNF648
BCL11B	DCT	FOXI2	IQCH	MBNL2	PARD3B	SAMD5	TICAM2	TMC1
BMPER	DEPTOR	FOXO1	IQSEC3	MDFIC	PARM1	SCG2	TLE1	TMEM132B
